# Barriers and facilitators to health insurance enrolment among people working in the informal sector in Morogoro, Tanzania

**DOI:** 10.12688/aasopenres.13289.1

**Published:** 2021-09-01

**Authors:** Elisante Abraham, Cindy Gray, Adeniyi F. Fagbamigbe, Fabrizio Tediosi, Brianna Otesinky, Joke Haafkens, Grace Mhalu, Sally Mtenga

**Affiliations:** 1 School of Life Sciences and Bio-engineering, The Nelson Mandela African Institution of Science and Technology, Arusha, Tanzania; 2 Institute of Health and Wellbeing, University of Glasgow, Bagamoyo, Tanzania; 3 School of Social and Political Sciences, Institute of Health and Wellbeing, University of Glasgow, Glasgow, UK; 4 College of Medicine, University of Ibadan, Ibadan, Nigeria; 5 The African Academy of Sciences (AAS), Nairobi, Kenya; 6 Swiss Tropical and Public Health Institute (Swiss TPH), Basel, Switzerland; 7 Amsterdam Institute of Advanced Labour Studies, University of Amsterdam, Amsterdam, The Netherlands

**Keywords:** health insurance, iCHF, enrolment, informal sector, local women food vendors, Bodaboda drivers, facilitators, barriers

## Abstract

**Background:** Health insurance is a crucial pathway towards the achievement of universal health coverage. In Tanzania, health-financing reforms are underway to speed up universal health coverage in the informal sector. Despite improved Community Health Fund (iCHF) rollout, iCHF enrolment remains a challenge in the informal sector. This study aimed to explore the perspectives of local women food vendors (LWFV) and *Bodaboda* (motorcycle taxi) drivers on factors that challenge and facilitate their enrolment in iCHF.

**Methods:** A qualitative study was conducted in Morogoro Municipality through in-depth interviews with LWFV (n=24) and *Bodaboda* drivers (n=26), and two focus group discussions with LWFV (n=8) and *Bodaboda* drivers (n=8). Theory of planned behaviour (TPB) constructs (attitude, subjective norms, and perceived control) provided a framework for the study and informed a thematic analysis focusing on the barriers and facilitators of iCHF enrolment.

**Results:** The views of LWFV and *Bodaboda* drivers on factors that influence iCHF enrolment converged. Three main barriers emerged: lack of knowledge about the iCHF (attitude); negative views from friends and families (subjective norms); and inability to overcome challenges, such as the quality and range of health services available to iCHF members and iCHF not being accepted at non-government facilities (perceived control). A number of facilitators were identified, including opinions that enrolling to iCHF made good financial sense (attitude), encouragement from already-enrolled friends and relatives (subjective norms) and the belief that enrolment payment is affordable (perceived control).

**Conclusions:** Results suggest that positive attitudes supported by perceived control and encouragement from significant others could potentially motivate LWFV and *Bodaboda* drivers to enroll in iCHF. However, more targeted information about the scheme is needed for individuals in the informal sector. There is also a need to ensure that quality health services are available, including coverage for non-communicable diseases (NCDs), and that non-government facilities accept iCHF.

## Introduction

In 2005, the World Health Assembly called for universal health coverage and defined it as securing access to adequate health care for all at an affordable price ^1^. Half of the world’s population cannot afford essential health services ^2^. Each year, around 150 million people suffer from financial catastrophe such as unemployment, selling of land and other properties, meaning they are unable to meet their needs; of these 150 million,100 million end up in poverty due to high out-of- pocket expenses for health care services ^3^.

Most low and middle-income countries (LMICs) in Sub-Saharan Africa (SSA) have embarked on health system reforms aimed at achieving universal health coverage to ensure the whole population, including those working in the informal sector, has access to good quality health services through pre-paid financing ^4^. The objective of universal health coverage is also reflected in the UN Sustainable Development Goal 3: “to ensure healthy lives and promote well-being for all at all ages” ^5^. Health insurance schemes have been implemented in several LMICs to reduce high out-of-pocket expenses for health care services among their members through raising revenue, pooling funds, and purchasing services ^6^.

Since becoming independent in 1961, Tanzania has been making efforts to ensure high quality, accessible and affordable health services for all citizens. Many reforms, including the introduction of cost-sharing in 1993, have been made to the health system financing structure ^7^. Under the cost-sharing policy, everyone with the ability to pay is required to contribute to the cost of health services, except specific groups, such as children under five and the elderly, who are exempt. The introduction of cost-sharing was followed by the establishment of prepayment schemes, starting with the Community Health Fund (CHF), which was piloted from 1996 in one district before a national CHF law was enacted five years later. The National Health Insurance Fund (NHIF) was also established in 1999 for government employees ^8^. From 2001, CHF was implemented at the level of the community (where it also targeted people working in the informal business sector ^9^) before it was reformed into the improved Community Health Fund (iCHF) in 2010. The iCHF is a voluntary health insurance scheme meant to compliment the NHIF, characterized by community members pooling funds to offset the cost of healthcare ^9^.

Prepayment health financing schemes in Tanzania are constantly challenged by low uptake, low coverage and sustainability issues ^10^; for example, the coverage of the iCHF is only 25% nationally ^11^. Previous studies of the CHF and iCHF indicate that the amount and timing of annual payments, the ability of the health insurance schemes to sustain service provision, accessibility of facilities, marketing and promotion strategies are among the factors influencing iCHF utilization in Tanzania ^12– 17^. Those with low income, including people working in the informal sector, have a limited capacity to afford enrolment into health insurance schemes. This group also has poor resilience to cope with health care needs during emergencies or shocks ^18^, the latter including heart attacks and serious injury or illness. Thus, iCHF enrolment among those working in the informal sector is crucial, as informal workers are also exposed to risky environments for contracting diseases or acquiring disabilities. However, little research on the factors that impede and facilitate uptake of iCHF insurance has targeted people working in the informal sector, such as local women food vendors (LWFV) and *Bodaboda* (motorcycle taxi) drivers.

This study aims to address this knowledge gap by investigating the barriers and facilitators to iCHF enrolment among LWFV and *Bodaboda* drivers in one district, Morogoro Municipality, Tanzania. As Tanzania is moving towards a single (mandatory) health insurance scheme ^9^, this study will help policymakers make informed choices and evidence-based decisions to support equity in access to health insurance and thus achieve universal health coverage.

## Methods

### Design

**Figure 1.**
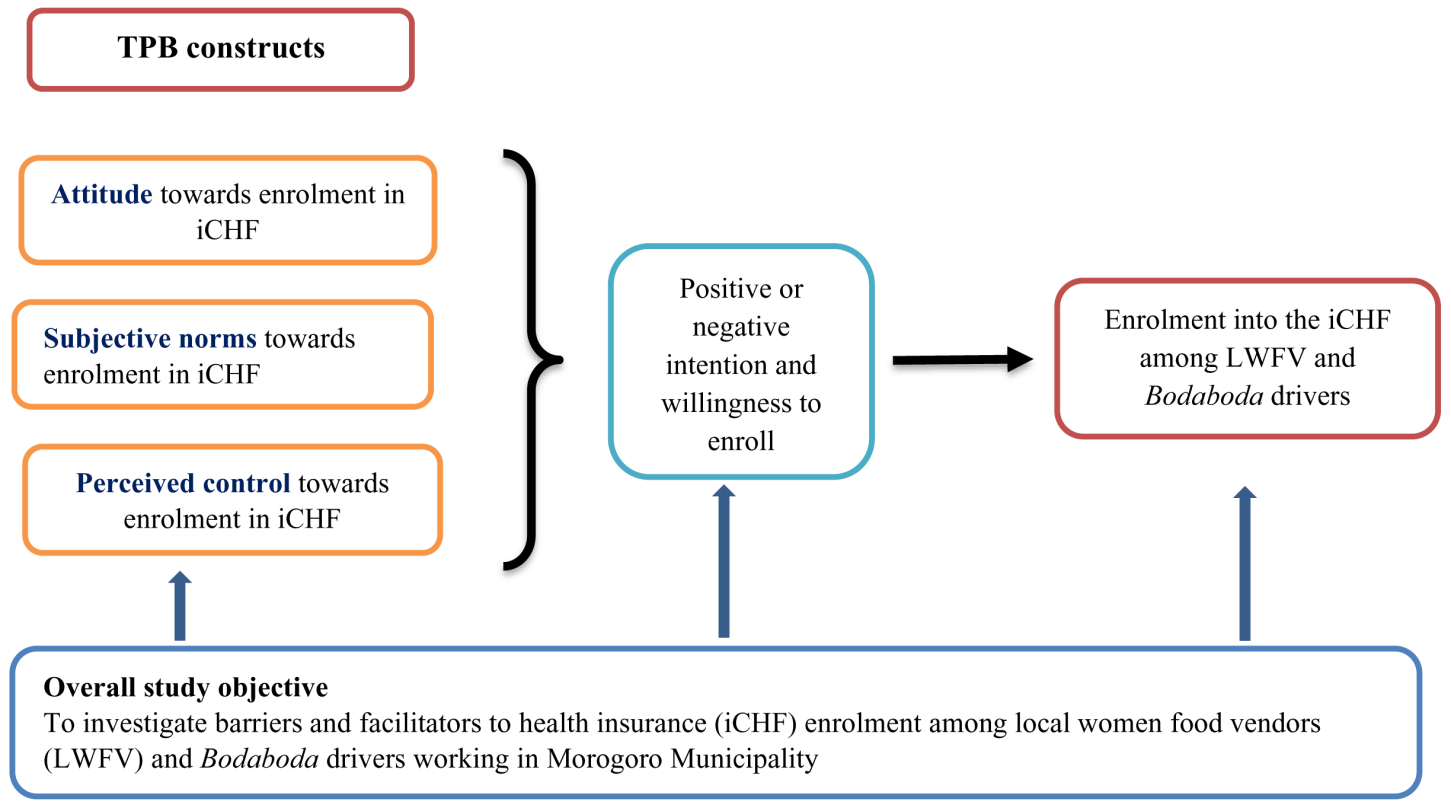
Conceptual framework of the assessment of factors that influence enrolment in health insurance.

A qualitative study was used to identify the barriers and facilitators to iCHF enrolment among LWFV and *Bodaboda* drivers living in Morogoro Municipality ^19^. Data were collected via semi-structured in-depth interviews (IDIs) and focus group discussions (FGDs). In qualitative research, multiple methods of data collection are a recognized way of ensuring the robustness of the findings (i.e., through triangulation) ^20^. All IDIs were completed before the FGDs and emerging findings from the IDIs informed the FGD guide. The theory of planned behaviour (TPB) informed the construction of the topic guides for both the IDIs and FGDs ^21^. TPB is a social psychological theory often applied in studies of health decision-making behaviors ^19– 28^, and suggests that behavioral intention is influenced by three main constructs: attitude, subjective norms and perceived behavioral control. In the current study, individual positive or negative opinions (attitude), the influence of significant others in behavior practices and choices (subjective norms), and personal belief in one’s ability to overcome barriers to engaging in the recommended behavior practices (perceived control) were explored to understand the barriers and facilitators of enrolment in iCHF among LWFV and *Bodaboda* drivers ( Figure 1).

### Setting

Data collection was conducted between July 2020 and September 2020 in thirteen wards of Morogoro Municipality. The town has a population of 315,866 ^29^ and was among the first regions to adopt iCHF in Tanzania in 2016. The Municipality is the headquarters of the Morogoro region where most of the administrative and socio-economic activities are implemented. The main types of employment opportunities in the Municipality are transportation, small-scale animal and bird keeping and other small businesses, which include LWFV and *Bodaboda* drivers. The population of the Municipality is heterogeneous with representation from almost all tribes across Tanzania; however, the main ethnic group is Waluguru ^30^. Health facilities range from local dispensaries and health centers to the regional referral hospital ^31^.

### Participants’ selection and recruitment

Purposive sampling was used to select 50 IDI participants (n=24 women and n=26 men), and 16 participants (n=8 women and n=8 men) for two FGDs. All women were LWFV and all men were *Bodaboda* drivers. The IDIs participants were selected from 13 out of the 29 wards in the Municipality, while FGD participants were selected from one of the 13 IDI wards. Our selection criteria ensured that single and married people of working age (18–59 years) were included. We also sampled both people who were enrolled and not enrolled in the iCHF. All participants were recruited on a face-to-face modality with the help of local government leaders, and LWFV and *Bodaboda* driver leaders. Before recruitment, leaders were informed about the purpose of the study and selection criteria to inform their approaches to potential participants.

### Data collection

Two different, but matched topic guides (one for LWFV and one for *Bodaboda* drivers), containing semi-structured questions and probes based on TPB constructs were used to conduct the interviews. Participant characteristics were collected at the start of the IDI or FGD. Before fieldwork, the IDI guides were piloted in one of the selected wards by the principal investigator (EA), and adaptations were made. The interviews were conducted by EA. At the time of this study, EA held a bachelor’s degree in sociology, and was pursuing a Master of Science in Public Health Research and worked as a Social Welfare Officer at Morogoro District Council, where he had also supervised the iCHF scheme from 2016 to 2018. The general assumption of the interviewer was, based on the TPB, that attitudes, subjective norms and perceived control may influence enrolment into iCHF. The specific assumptions were that factors such as lack of awareness, poor income, and poor health care at facilities may hinder enrolment into iCHF among individuals working in the informal sector. To improve interactions and avoid potential gender hierarchies, we held separate FGDs for men and women. All IDIs and FGDs were conducted in Kiswahili language in private locations that were chosen by the participants as convenient relative to their place of work. The IDIs were conducted until information saturation was achieved and no new insights emerged. Both the interviews and FGDs took about 30 to 35 minutes, while field notes were taken during the sessions. All IDIs and FGDs were recorded digitally with participant consent.

### Data analysis

The audio recordings were transcribed verbatim. EA initially read each transcript line-by-line to gain an initial impression of the insights that emerged from participants’ narratives. A deductive approach was used to identify themes based on TPB constructs (Attitudes towards iCHF enrolment; the influence of subjective and other social norms on iCHF enrolment; and perceived control of enrolment into the iCHF). An inductive approach was used to identify strategies that might be used to improve LWFV and *Bodaboda* drivers’ enrolment into the iCHF. An open coding framework was developed by EA and, with support from SM, the codes were grouped into barriers and facilitators of iCHF enrolment ( Figure 1) and strategies to promote enrolment of LWFV and *Bodaboda* drivers into the iCHF. All data were analyzed in Kiswahili language. Themes were compared between LWFV and *Bodaboda drivers*. Four people who had participated in the IDIs (one man, one woman) and FGDs (one man, one woman) checked the themes for accuracy ^32^.

### Ethical approval and consent to participate

The study was reviewed and approved by the Ifakara Health Institute ethics committee (approval number IHI/IRB/No: 30-2020). All participants provided written informed consent. Participants were assured confidentiality and informed about their right to withdraw from the study at any time.

## Results

A total of 66 people (34 males and 32 females) participated in the study. As Table 1 shows, their age ranged from 18 to 59 years and most (66%) had at least a primary-level education. About one-third had attended secondary education. Only a few participants (24%) were enrolled in iCHF. The majority were married with one to five children. More than half were Christians and had lived in the study area for more than two years. 

**Table 1.** Summary of participant characteristics (IDIs N=50, FGDs N=16); IDIs: in-depth interviews, FGDs: focus group discussions.

Participant Characteristics	IDIs (N)	%	FGDs (N)	%
**Sex** Male Female	26 24	52.0 48.0	8 8	50.0 50.0
**Age** 18-25 26-35 36-45 45-59	8 22 13 7	16.0 44.0 26.0 14.0	3 7 4 2	18.8 43.7 25.0 12.5
**Education** Never attended school Primary level Secondary level Higher level	0 33 15 2	0.0 66.0 30.0 4.0	0 9 7 0	0.0 56.3 43.7 0.0
**Other employment** With other occupation Without other occupation	19 31	38.0 62.0	6 10	37.5 62.5
**Health insurance status** Insured Not insured	12 38	24.0 76.0	4 12	25.0 75.0
**Marital status** Married Divorced Widowed Never married	30 1 2 17	60.0 2.0 4.0 34.0	10 1 1 4	62.5 6.3 6.2 25.0
**Number of children** 0 1-2 3-5 > 5	8 20 19 3	6.0 40.0 8.0 >6.0	3 6 5 2	8.8 37.5 31.2 12.5
**Religion** Christian Muslim	29 21	58.0 42.0	10 6	62.5 37.5
**Time living in area** < 1 year 2-5 years 5-10 years >10 years	8 12 16 14	16.0 24.0 32.0 28.0	3 4 5 4	18.8 25.0 31.2 25.0

### Barriers to enrolment in the iCHF

The analysis revealed several barriers to iCHF enrolment in relation to the TPB constructs of attitude, subjective norms and perceived control, most of which were common among LWFV and *Bodaboda* drivers. These barriers are explored in detail in the following sections.

***Lack of knowledge about the iCHF.*** An important attitudinal barrier reported by participants (particularly those who were not insured) was the view that there was limited awareness of the iCHF scheme among informal workers. Both LWFV and *Bodaboda* drivers highlighted that a lack of information about the scheme, including where to enroll, how to enroll and the cost of enrolment, prevented some people from signing up, even when they were motivated and encouraged by others to do so.

*“There is a lack of knowledge among most of us about this scheme [iCHF]. Some of us have money but we do not understand well about health insurance. We have not been provided with enough education, so it will be difficult for us to join” [IDI 07, LWFV, uninsured]*

     “ *The community motivates me to enroll in iCHF, but what makes me not to enroll is that I don’t know where to go for enrolment [...] [FGD 05, Bodaboda driver, uninsured].*

This knowledge deficit was felt to be largely due to the fact that iCHF providers overlooked informal workers like LWFV and *Bodaboda* drivers when it came to information campaigns. As one *Bodaboda* driver explained, there was a widespread view that more should be done about targeting iCHF promotion materials to informal workers. 

*“…we [Bodaboda drivers] have not been given education about that insurance, so it is difficult to enroll themselves in the insurance [iCHF]. If one would have received the education or knows the benefits and the risk [of not enrolling in iCHF] they would have enrolled themselves” [FGD 01, Bodaboda driver, uninsured]*

Another attitudinal barrier identified by participants was the fact that for some people, iCHF was seen as a low priority in relation to financial decision-making. One LWFV explained that despite having sufficient income, some people choose to spend their money on other things, rather than health insurance.

*“What I see is carelessness in decision making. A local woman food vendor cannot fail to get thirty thousand shillings per year. So, it is carelessness in decision making! We are used to being careless but if we’re motivated, we can enroll. Someone may have a good income but keeps saying ‘I will go’ and then she ignores it.” [IDI 39, LWFV, uninsured]*

***Negative views from friends and families.*** Such reluctance to enroll may in part reflect negative subjective norms towards the iCHF. Some participants described how stories from friends and family members, including poor quality of health care, had put them off enrolling in the iCHF scheme. For example, one LWFV said she had heard that health facilities withheld some treatment options from iCHF patients.

*“Our friends and relatives have been uninspired to enroll in the health insurance due to the nature of care provided to those enrolled. They [friends and relatives] told us that those with iCHF cards get disturbance in getting specific health care such as drugs. Sometimes they’re told their insurance does not cover all the services” [IDI 17, LWFV, uninsured]*

***Inability to overcome challenges.*** Three contextual and structural barriers that were viewed as being beyond the personal control of participants were also reported. First, further concerns around quality of health care emerged because iCHF was only accepted in government health facilities. As one LWFV complained, this meant that the (perceived) better health services in local private facilities were not available to iCHF holders. 

*“iCHF insurance is not accepted in private hospitals and you find that these facilities are close to us, they also provide good services, but people don’t go there because their services are expensive and they don’t accept these small insurances [iCHF]. This discourages us… and it is beyond our capacity. It is the government that needs to solve this” [IDI 03, LWFV, uninsured]*

A second issue raised was the fact that the iCHF does not cover treatment for non-communicable diseases (NCDs). One LWFV, who already had insurance, complained it was unfair that she still had to pay for treatment for her heart disease.

*“That insurance [iCHF] does not cover these diseases like diabetes and hypertension. I am also a victim of chronic diseases and heart problems but when I go to the hospital with an insurance card the only help that I get is just registration and doctor consultation, and I need to pay for the rest. When I take a doctor’s drug sheet to the pharmacy, I don’t get even a single drug, so I go to buy outside the hospital…the government should work on this” [FGD 10, LWFV, insured]*

A third barrier reported by *Bodaboda* drivers was that the ‘on-demand’ nature of their work made it hard for them to go to iCHF offices during opening hours. They further stressed that their long working days (from early morning through the night) made it almost impossible for them to enroll in the scheme, but one suggested that this problem might be overcome if iCHF providers were able to come to their place of work. 

*“I can say that for us,* Bodaboda *drivers, time is a problem. I can’t leave my job to go for enrolment! I wish they [iCHF registrars] would be going to all places where we park our motorcycles, just like how you are doing now and tell them what to do for enrolment and enroll them at their stations” [IDI 21, Bodaboda driver, uninsured]*

### Facilitators to enrolment in iCHF

The main barriers to enrolment in iCHF identified by participants therefore included lack of knowledge about the schemes available, negative stories about iCHF from friends and family, concerns over the quality of health care and the services available, and lack of accessibility. However, many participants, particularly those who were insured, were also able to identify factors that might facilitate iCHF uptake locally. These are now explored in relation to the three TPB constructs.

***Value for money.*** Participants who were already insured exhibited an extremely positive attitude towards the iCHF. Many felt that enrolling made good financial sense. One *Bodaboda* driver described how the iCHF helped people get the healthcare they needed when they did not have ready access to cash.

*“There are many benefits [...] whenever you face health problems, you do not need to wait until the next day or to find money, you simply visit a facility and get instant service at low costs” [IDI 14,* Bodaboda *driver, insured]*

Interestingly, some people drew parallels between the iCHF and a savings scheme. One *Bodaboda* driver told how he viewed it as a way of investing money in his health. 

*“Health insurance [iCHF] is something good! Those who introduced it thought very well because you can have something [money] today and tomorrow you do not have it. So, it acts like savings for your health since health is vital in human life” [IDI 41, Bodaboda driver, insured]*

The same driver described how the upfront investment gave him peace of mind in knowing he would be able to access any health services he required as and when he needed them.

*“Benefits are many; first, you are ensured of your health safety at all times, second, even when you have no money, you can access treatment easily at hospital” [IDI 41,* Bodaboda *driver, insured]*

Importantly, most insured participants suggested that the annual payment was well within many people’s personal capacity to pay. For example, one LWVF described her iCHF as excellent value for money, and suggested that it was something everyone should consider.

*“In my opinion,… thirty thousand shillings [13 USD] is a very small amount to support health care [iCHF] for the whole year for more than five people [in the family]. Imagine you and your children-a total of six! This is affordable for someone with a low income. Even for me, a local woman food vendor. The affordability of the iCHF surpasses all other challenges associated with the iCHF. Sometimes it is only stubbornness that makes people not to join the insurance” [IDI 03, LWFV, insured]*

***Encouragement from already-enrolled friends and relatives.*** In contrast to the negative subjective norms noted by some participants in relation to iCHF, other participants described the enthusiasm of friends and relatives who were already enrolled in the scheme. It was clear from the accounts given that these recommendations from trusted people could be extremely persuasive.

*“…the way I see them [friends and relatives], their response is very positive. The response is there and it also motivates us to join the scheme! But just like I told you earlier, we are not aware of where to start or end although people desire that thing [enrolling to insurance] very much” [IDI 05, Bodaboda driver, uninsured]*

*“I see that the response of friends and relatives in joining the scheme [iCHF] is high. The response is there, a big one! which also encourages me to join the scheme” [IDI 32, LWFV, uninsured].*

Finally, despite the concerns about the quality of healthcare available to iCHF holders raised by some participants without insurance in the previous section, one LWFV who had been enrolled in iCHF for some years expressed her satisfaction with the treatment she had received through the scheme.

*“Truly, health insurance [iCHF] is super! Personally, it is now about seven years since we were insured, and when we get any [health] problem, we simply go to any good hospital in town just with bus fare and get attended well. So, I see it is very beneficial” [IDI 07, LWFV, insured]*

### Promoting uptake of iCHF 

As well as making iCHF enrolment more accessible to informal workers (as reported above), in order to encourage more widespread iCHF uptake, participants were clear that more initiatives were needed to promote the benefits and affordability of the scheme. Some called on iCHF providers to run targeted awareness-raising events locally:

*“They should try their best to conduct seminars. They should go to public gatherings and educate people on health insurance. Income is not a problem to some people, they lack the knowledge” [IDI 27, LWFV, uninsured]*

However, others felt that the quality and range of health care and health facilities available to iCHF holders would have to be improved to persuade people to enroll.

*“What’s important is to improve iCHF services at the facilities because people are getting discouraged by the current situation. The government should set special units, care providers and drugs for the iCHF members” [FGD 14, LWFV, insured]*

Importantly, participants called for transparency within the iCHF system. Some stressed the importance of having a robust monitoring and evaluation framework to quality-assure iCHF implementation and delivery of health care to insured patients within local health facilities. A *Bodaboda* driver suggested that one way of doing this might involve representatives from the iCHF providers performing regular spot checks to assess the scheme’s operation at each facility.

*“I advise them [iCHF implementers] to make follow-up of health insurance operations so as to know how things are implemented. That is to see if iCHF is properly utilized as required or it is provided as intended, because one can go to the health facility and find nothing and he has already paid for insurance” [IDI 25, Bodaboda driver, uninsured]*

## Discussion

This study aimed to explore the perspectives of LWFV and *Bodaboda* (motorcycle taxi) drivers on factors that challenge and facilitate their enrolment in iCHF. The main barriers identified included: lack of knowledge about the iCHF (attitude); negative views from friends and families (subjective norms); and inability to overcome challenges such as the (poor) quality and (limited) range of health care services available to iCHF members, and inability to access iCHF enrolment (perceived control) *.* The main facilitators included views that iCHF enrolment made good financial sense (attitude) and was affordable (perceived control), and encouragement from already-enrolled friends and relatives (subjective norms) *.*

This study suggests that iCHF has gained recognition among LWFV and *Bodaboda* drivers despite some implementation challenges. Many LWFV and *Bodaboda* drivers appeared to appreciate the benefits of the scheme, which is an important motivational factor for enrolment and re-enrolment. Our findings are consistent with previous studies from Ethiopia reporting that health insurance was viewed as better than out-of-pocket payments for health care ^33, 34^. Likewise in Kenya, health insurance was perceived to have many advantages, such as providing financial protection to members and making them feel at ease when their relatives were in hospitals ^35^; and a study from Ghana revealed that community members saw the National Health Insurance Scheme (NHIS) as a form of protection from potentially catastrophic healthcare payments ^36^. 

The current study suggests that despite some negative opinions, many people who are already enrolled in the iCHF are extremely positive about it and that recommendations from trusted people could motivate LWFV and *Bodaboda* drivers to sign up. This finding is consistent with a study in Ghana ^37^ which revealed that close relatives positively influenced enrolment and re-enrolment in the National Health Insurance Scheme (NHIS). Likewise, research in rural Uganda reported that having a neighbor enrolled in a Community Based Health Insurance (CBHI) scheme increased the likelihood of membership renewal ^38^. Involving those already enrolled in the scheme to describe their experiences during awareness campaigns may therefore help to promote iCHF uptake among informal workers. On the other hand, if relatives, friends and neighbours have a negative view of the iCHF, this may adversely impact enrolment.

An important barrier to iCHF enrolment among LWFV and *Bodaboda* drivers appeared to be low knowledge about the iCHF scheme, including where and how to enroll, and the cost of enrolment. Limited information about iCHF implementation represents a missed opportunity towards achieving universal health coverage in Tanzania since this may deter informal sector workers from joining the scheme, even when, as participants who were already enrolled made it clear, the annual payments are affordable. These findings are consistent with a study from Ghana where some community members said that they had not heard of any scheme operating in their area ^36^. Similarly, in rural South-western Uganda, households with limited access to information such as how much they need to pay, had a higher likelihood of not enrolling or renewing their health insurance membership than those who were better informed ^38^. Other studies from Ethiopia also suggested a positive association between information and enrolment/re-enrolment in health insurance schemes ^39, 40^.

Our study found that perceptions of poor quality and limited health care options for iCHF members can demotivate people to enroll. This is consistent with a systematic review of health insurance in LMICs which indicated that poor satisfaction with health services dissuaded people from joining schemes and led to discontinuation of memberships ^41^. Lack of coverage of NCDs emerged as a particular concern, and other research has found that people are motivated to enroll when a health insurance includes NCD coverage ^40^. These findings are important as universal health coverage aims to ensure access to health care for all people regardless of their socio-economic backgrounds and health condition, and iCHF is one way to provide this. A systematic review reported that good quality health services can attract more vulnerable people to enroll in health insurance ^41^. Therefore, concerns among informal sectors workers about the quality and coverage of the services available will impede optimal use of health insurance schemes and the achievement of better health for all in Tanzania.

In addition to improving the quality of health care available, other suggestions to increase iCHF enrolment among LWFV and *Bodaboda* drivers included running local awareness-raising campaigns and bringing the enrolment procedures to their places of work. A study in Southern Nigeria provided similar recommendations that enrolment can be improved through awareness-raising and bringing enrolment services to the people ^42^. Enhancing monitoring of how iCHF is being implemented at health facilities was also recommended by participants, which echoes a study in Kenya reporting that health insurance providers should be able to demonstrate proper financial management and monitoring of healthcare services ^35^.

This study had a number of strengths. These included the high number of interviews conducted to ensure data saturation was achieved, the purposive sampling strategy adopted to provide diversity of perspectives, and the use of both IDIs and FGDs and participant checking to ensure the findings were robust. However, one important limitation was that the study was only conducted in one setting, Morogoro Municipality, meaning the findings may not be generalizable to other (and particularly rural) settings.

## Conclusions

The study suggests that an overall positive attitude towards iCHF, recognition of the benefits of iCHF and encouragement from significant others, could support enrolment among informal sector workers in Tanzania. However, awareness-raising campaigns targeting LWFV and *Bodaboda* drivers (including the experiences of local role models) and initiatives to make enrolment easily accessible to them are required to improve their uptake of iCHF. There is also a need to ensure that good quality health care is available to iCHF members, which includes extending coverage to include NCDs and non-government health facilities.

## List of abbreviations

CBHI……………………………………………………..Community-Based Health Insurance

CHF………………………………………………………Community Health Fund

FGDs……………………………………………………..Focus Group Discussions

iCHF……………………………………..……………….improved Community Health Fund

IDIs……………………………………………………….In-Depth Interviews

IHI……………………………………… ……………….Ifakara Health Institute

IRB………………………………………………………..Institutional Review Board

LMICs…………………………………………………….Low and Middle Income Countries

LWFV…………………………………………………… Local Women Food Venders

NCDs……………………………………………………..Non-Communicable Diseases

NHIS……………………………………………………...National Health Insurance Scheme

NHIF………………………………………………….......National Health Insurance Fund

TPB ………………………………………………………Theory of Planned Behavior

## Data availability

### Underlying data

The Institutional Review Board (IRB) of the Ifakara Health Institute does not allow the transcript data from the in-depth interviews or the focus group discussions to be made publicly available, due to the fact that policy for data circulation has to be signed. Interested researchers should contact the corresponding author or irb-submission@ihi.or.tz. Data access will be granted under the following conditions: (i) signing the data sharing agreement; (ii) waiver of the informed consents by the IRB if the justifications are considered to be ethically and scientifically sound, since the consent forms clearly stated that the participants’ data would not be shared outside the Institute.

### Extended data

Figshare: Barriers and facilitators to health insurance enrolment among people working in the informal sector in Morogoro, Tanzania, https://doi.org/10.6084/m9.figshare.15161349 ^43^.

This project contains the following extended data:

- IDI questions for local women food vendors
- IDI questions for *Bodaboda* drivers
- FGD Guide for local women food vendors
- FGD Guide for *Bodaboda* drivers

Data are available under the terms of the Creative Commons Zero “No rights reserved” data waiver (CC0 1.0 Public domain dedication).
