# Supplementary material for: Barriers and facilitators to health insurance enrolment among people working in the informal sector in Morogoro, Tanzania
Source: AAS Open Res. 2021 Sep 1;4:45. [Version 1] doi: 10.12688/aasopenres.13289.1 (PMC11231626; doi:10.12688/aasopenres.13289.1)
Supplement: Peer review history

# Reviewer report: Reviewer response for version 1

**DOI:** 10.21956/aasopenres.14413.r29603

**Published:** 2023-07-18

**Authors:** Dossè M. DJAHINI-AFAWOUBO

**Affiliations:** 1 Department of economics, Université de Lomé, Lomé, Togo

This study aimed to explore Barriers and facilitators to health insurance enrolment among people working in the informal sector (local women food vendors (LWFV) and *Bodaboda* (motorcycle taxi) drivers) in Morogoro, Tanzania. This subject is important, particularly in Africa where a large part of the population works in the informal economy. The study could help policymakers make informed choices and evidence-based decisions to support equity in access to health insurance and thus achieve universal health coverage. However, I provide some suggestions below.

1. In the introduction section, the authors should show the contributions of the article. The authors argue that little research on the factors that impede and facilitate uptake of iCHF insurance has targeted people working in the informal sector, such as local women food vendors (LWFV) and *Bodaboda* (motorcycle taxi) drivers. These previous studies should be cited. In addition, the authors should show how this article addresses the limitations of these previous studies.
2. The introduction section should also announce the structure of the article.
3. In the Participants’ selection and recruitment section, what motivated the selection of the 50 IDI participants? There should be a discussion about the sample size and its representativeness.
4. According to its title, the paper is about informal sector. However, none is said about informality in the paper. Are all motorcycle taxi drivers working in the informal sector in Tanzania? There should be a discussion on what informality refers to in the paper.
5. In the conclusion section, the authors should recall the objectives of the study, the methodological approach used, and the main results before making any recommendations. I suggest that each recommendation made by the authors be linked to a result of the study.

Is the work clearly and accurately presented and does it cite the current literature?

Yes

If applicable, is the statistical analysis and its interpretation appropriate?

Yes

Are all the source data underlying the results available to ensure full reproducibility?

Yes

Is the study design appropriate and is the work technically sound?

Yes

Are the conclusions drawn adequately supported by the results?

Partly

Are sufficient details of methods and analysis provided to allow replication by others?

Yes

Reviewer Expertise:

Health economics, education economics, Economic development

I confirm that I have read this submission and believe that I have an appropriate level of expertise to confirm that it is of an acceptable scientific standard.

# Response

**Published:** 2024-03-13

**Authors:** Elisante Abraham

**Affiliations:** The Nelson Mandela African Institution of Science and Technology, Tanzania

**Introduction**

 > In the introduction section, the authors should show the contributions of the article. The authors argue that little research on the factors that impede and facilitate uptake of iCHF insurance has targeted people working in the informal sector, such as local women food vendors (LWFV) and Bodaboda (motorcycle taxi) drivers. These previous studies should be cited. In addition, the authors should show how this article addresses the limitations of these previous studies.

 - Thank you for this suggestion. Kindly note that the contribution of the article is placed at the last but one paragraph of the introduction section. Refer to page 4. - This is appreciated. By saying ‘little research’ we meant that there is limited information related to that evidence. However, citations of previous studies which were conducted on the other community health funds (not iCHF) have been provided. Refer to page 3. - Also, explanation on how this article addresses the limitations of these previous studies have been shown. Refer to page 3. 

 > The introduction section should also announce the structure of the article.

 - It is now indicated that this work is divided into five sections which are the introduction, methods, results, discussion and conclusions. Refer to page 5.  

 > In the Participants’ selection and recruitment section, what motivated the selection of the 50 IDI participants? There should be a discussion about the sample size and its representativeness.

 - Based on an estimated saturation point, purposive sampling was used to select and recruit 50 IDI participants; this was achieved while conducting data collection i.e. information saturation was achieved at the 50th interview.  

 > According to its title, the paper is about informal sector. However, none is said about informality in the paper. Are all motorcycle taxi drivers working in the informal sector in Tanzania? There should be a discussion on what informality refers to in the paper.

 -Thank you. Kindly be informed that the informality in the paper is presented by the fact that iCHF is meant for those employed in the informal sector (LWFV and motorcycle taxi drivers sampled). These two (the iCHF and the study group) have both being discussed throughout the paper. - We would also like to bring into your awareness that in Tanzania, all *bodaboda* drivers (motorcycle taxi drivers) work in the informal sector. So when one mentions “ *bodaboda* driver” this simply means the referred is not employed in the formal sector.  

**Conclusion ** In the conclusion section, the authors should recall the objectives of the study, the methodological approach used, and the main results before making any recommendations. I suggest that each recommendation made by the authors be linked to a result of the study.

 - This comment is appreciated. The conclusion has been rephrased and recommendations are modified to link to results of the study.            

# Reviewer report: Reviewer response for version 1

**DOI:** 10.21956/aasopenres.14413.r28872

**Published:** 2021-10-01

**Authors:** Lauren Suchman

**Affiliations:** 1 Institute for Global Health Sciences, University of California, San Francisco, San Francisco, CA, USA

Introduction

- You note that SDG 3 is “to ensure healthy lives,” but you might want to note that SDG 3.8 specifically calls for UHC.
- The literature review provides helpful context, but could benefit from some exploration of the literature on similar initiatives outside of Tanzania. For example, Kenya has implemented an NHIF scheme that specifically targets the informal sector – what have the opportunities and challenges been there? How successful has a community-based insurance scheme been in other countries similar to Tanzania? What kinds of challenges have countries like Ghana, with the continent’s longest-running national health insurance scheme, faced when trying to provide health insurance coverage for their entire population?

Methods

- Design
  - In the U.S., we would interpret the phrase “significant others” as romantic partners. So, you might want to re-phrase this here and in the Conclusion as well if you expect a U.S. audience at all.
- Data collection
  - Please provide a high-level overview of the structure and content of the guides (i.e. section titles) and examples of some of the questions that were asked
  - How did you know saturation was achieved while conducting data collection? Was analysis simultaneous with data collection? 

Results

- You say that participants had a lack of “awareness” about the iCHF scheme, but then say they have a lack of “information.” If they lack awareness, this suggests to me that they don’t know about the scheme at all, but I think you mean they lack information regarding where and how to enroll.
- Negative views from friends and family
  - It would be helpful to have more information here. I would suggest summarizing common themes that come up when people say they have heard bad things from others so the reader knows what kinds of complaints are circulating. It would also be helpful to have some more contextual information here. What do the participants themselves think of these complaints? What makes them seem believable or especially relevant? In other words, why would these complaints seem like a good reason to forego health insurance?
- Inability to overcome challenges
  - I’m not sure that your point about NCDs works because the quote you use is from a woman who is insured. So, even though she complains that she can’t use her iCHF coverage for her heart disease, this isn’t a barrier to enrollment for her because she is still enrolled. Did others say they hadn’t enrolled because they were concerned that NCDs weren’t covered, or is this more of a challenge for those who are already in the system?

Discussion

- This section provides a nice overview of key findings and an engagement with relevant literature, but it would be nice to have some discussion of how policymakers might act on your findings, particularly in the case of barriers you have identified. For example, in the Results section you quote a Bodaboda driver who suggests more sensitization for himself and his fellow drivers. You mention a study from Nigeria below, but it would be nice to have more information on what this program looked like. How is sensitization best done? What makes a good campaign successful? How might other concerns about quality and limited coverage be addressed?

Is the work clearly and accurately presented and does it cite the current literature?

Partly

If applicable, is the statistical analysis and its interpretation appropriate?

Not applicable

Are all the source data underlying the results available to ensure full reproducibility?

No

Is the study design appropriate and is the work technically sound?

Yes

Are the conclusions drawn adequately supported by the results?

Yes

Are sufficient details of methods and analysis provided to allow replication by others?

Partly

Reviewer Expertise:

Qualitative research, health financing, Kenya, Ghana

I confirm that I have read this submission and believe that I have an appropriate level of expertise to confirm that it is of an acceptable scientific standard, however I have significant reservations, as outlined above.

# Response

**Published:** 2024-03-13

**Authors:** Elisante Abraham

**Affiliations:** The Nelson Mandela African Institution of Science and Technology, Tanzania

**Introduction** > You note that SDG 3 is “to ensure healthy lives,” but you might want to note that SDG 3.8 specifically calls for UHC.

 - Thank you for this comment, we have added the SDG 3.8 in the background section as well. Refer to page 3.  

**Literature review** > The literature review provides helpful context, but could benefit from some exploration of the literature on similar initiatives outside of Tanzania. For example, Kenya has implemented an NHIF scheme that specifically targets the informal sector – what have the opportunities and challenges been there? How successful has a community-based insurance scheme been in other countries similar to Tanzania? What kinds of challenges have countries like Ghana, with the continent’s longest-running national health insurance scheme, faced when trying to provide health insurance coverage for their entire population?

 -Thanks for enlightening on more similar literature on informal sector. We have added some of these new insights in our literature review section and background. Refer to page 3.

**Methods** **Design** > In the U.S., we would interpret the phrase “significant others” as romantic partners. So, you might want to re-phrase this here and, in the Conclusion, as well if you expect a U.S. audience at all.

 - It is interesting! However, the concept is derived from the theory of planned behaviour (TPB) as explained in the paper. We also added an elaboration that ‘significant others’ as used in our study context refers to ‘other people who have the potential to influence the behaviour of individuals; they include spouses, relatives, children, and friends.’  Refer to page 4.

**Data collection** > Please provide a high-level overview of the structure and content of the guides (i.e. section titles and examples of some of the questions that were asked). 

 - This is noted with thanks. We have added section titles and examples of some of the questions that were asked have been provided. Refer to pages 5.

 > How did you know saturation was achieved while conducting data collection? Was analysis simultaneous with data collection?

 - While conducting data collection, saturation was achieved when no new information was coming up from the interviews. As known, in qualitative research, analysis begins during data collection. As such, analysis was simultaneous with data collection.    

**Results** > You say that participants had a lack of “awareness” about the iCHF scheme, but then say they have a lack of “information.” If they lack awareness, this suggests to me that they don’t know about the scheme at all, but I think you mean they lack information regarding where and how to enrol.

 - Thanks for this observation. To the best of our knowledge, usually information leads to awareness. As such both aspects could be a possible reflection of participant’s narratives. ‘Our findings suggested that participants lacked information about iCHF as well as awareness about it’ Thus, it is lack of information and awareness as well.

**Negative views from friends and family** > It would be helpful to have more information here. I would suggest summarizing common themes that come up when people say they have heard bad things from others so the reader knows what kinds of complaints are circulating. It would also be helpful to have some more contextual information here. What do the participants themselves think of these complaints? What makes them seem believable o respecially relevant? In other words, why would these complaints seem like a good reason to forego health insurance?

 - More information has been added under “ **Negative views from friends and family**”. Common themes that come up when people say they have heard bad things from others are now summarized.

**Inability to overcome challenges** > I’m not sure that your point about NCDs works because the quote you use is from a woman who is insured. So, even though she complains that she can’t use her iCHF coverage for her heart disease, this isn’t a barrier to enrollment for her because she is still enrolled. Did others say they hadn’t enrolled because they were concerned that NCDs weren’t covered, or is this more of a challenge for those who are already in the system?

 - Thank you. The iCHF is renewable in an annual basis, and therefore a member such as this woman who can’t use her iCHF coverage for her heart disease is likely not to re-enroll. Also, there is a possibility of the unenrolled ones to decide not to enroll by hearing such experience.  

**Discussion** > This section provides a nice overview of key findings and an engagement with relevant literature, but it would be nice to have some discussion of how policymakers might act on your findings, particularly in the case of barriers you have identified. For example, in the Results section you quote a Bodaboda driver who suggests more sensitization for himself and his fellow drivers. You mention a study from Nigeria below, but it would be nice to have more information on what this program looked like. How is sensitization best done? What makes a good campaign successful? How might other concerns about quality and limited coverage be addressed?

 - As observed in the fifth paragraph of the discussion, other suggestions to increase iCHF enrolment among LWFV and *Bodaboda* drivers included running local awareness-raising campaigns. Also, under **Promoting uptake of iCHF **a suggestion is given to policy makers on how to best do the sensitization (awareness-raising campaigns). That is through conducting seminars; they should go to public gatherings and educate people on health insurance *.*                  
